# Domestic Ducks and H5N1 Influenza Epidemic, Thailand

**DOI:** 10.3201/eid1204.051614

**Published:** 2006-04

**Authors:** Thaweesak Songserm, Rungroj Jam-on, Numdee Sae-Heng, Noppadol Meemak, Diane J. Hulse-Post, Katharine M. Sturm-Ramirez, Robert G. Webster

**Affiliations:** * Kasetsart University, Nakornpathom, Thailand;; † Western Veterinary Research and Development Center, Rachaburi, Thailand;; ‡ St. Jude Children's Research Hospital, Memphis, Tennessee, USA

**Keywords:** H5N1 influenza, ducks, Thailand, closed house, backyard, open house, free range, synopsis

## Abstract

In addition to causing 12 human deaths and 17 cases of human infection, the 2004 outbreak of H5N1 influenza virus in Thailand resulted in the death or slaughter of 60 million domestic fowl and the disruption of poultry production and trade. After domestic ducks were recognized as silent carriers of H5N1 influenza virus, government teams went into every village to cull flocks in which virus was detected; these team efforts markedly reduced H5N1 infection. Here we examine the pathobiology and epidemiology of H5N1 influenza virus in the 4 systems of duck raising used in Thailand in 2004. No influenza viruses were detected in ducks raised in "closed" houses with high biosecurity. However, H5N1 influenza virus was prevalent among ducks raised in "open" houses, free-ranging (grazing) ducks, and backyard ducks.

## Abstract 2

Traditional methods of raising ducks in Southeast Asia must be modified.

The continuing spread of H5N1 avian influenza viruses from eastern Asia to domestic and wild birds in central Asian countries, including Mongolia, Kazakhstan, Russia, and Turkey, indicates the extent to which the geographic range of this highly pathogenic influenza virus has expanded. The highly pathogenic H5N1 viruses were first detected in 1996 in geese in Guangdong, China (1); they later spread to ducks in the coastal provinces of South China (2) and to Hong Kong's live poultry markets (3). These viruses infected at least 18 persons in Hong Kong, 6 of whom died (4). The viruses were eradicated in 1998 by the culling of all poultry in Hong Kong and by changing marketing practices. Although these particular genotypes have not been detected again, other H5N1 genotypes continued to emerge in 2000 and 2001 (5).

The biology of the H5N1 viruses changed dramatically for the first time in late 2002, when the viruses were isolated from dead wild aquatic birds in Hong Kong and from decorative waterfowl that died in Kowloon Park, Hong Kong (6,7). After the Z genotype of H5N1 influenza became established as the dominant H5N1 influenza virus in eastern Asia, it was transmitted to persons in Vietnam, Thailand, and Cambodia. In 2004, a distinguishable genotype was transmitted to persons in Indonesia (8). Most human cases have resulted from the direct transmission of virus from poultry to humans (9). To date, evidence for human-to-human transmission is limited (10,11). In Thailand, 13 persons infected with an H5N1 influenza virus died in 2004, and 2 additional human deaths occurred in October 2005. By contrast, in neighboring Vietnam, 42 human deaths caused by H5N1 influenza virus were reported in 2005. What accounts for these differences? Here we examine the hypothesis that the lower death rate in Thailand resulted in part from that government's recognition of the role of backyard chickens and domestic ducks in the spread and perpetration of H5N1 influenza virus and the government's aggressive culling of flocks in which the virus was detected (12).

Thai health officials recognized that the spread of H5N1 influenza viruses to domestic chickens correlated with the distribution of free-grazing ducks (13). At the beginning of the 2004 poultry outbreak, ducks were raised in 1 of 4 systems: 1) in high-biosecurity closed houses; 2) in moderately high-biosecurity open houses (ducks raised for meat and laying ducks); 3) in rice fields after harvest (free-range or so-called grazing ducks); or 4) in backyards (backyard ducks). We discuss each method, particularly emphasizing the role of grazing ducks in the perpetuation and spread of H5N1 in the country. We also describe the clinical and pathologic changes in ducks and consider the current policies regarding duck raising in Thailand. We conclude that the traditional methods of raising ducks in Thailand and the rest of Southeast Asia must be modified if we are to control the spread of avian influenza virus.

## Methods of Duck Raising in Thailand

**Figure 1:**
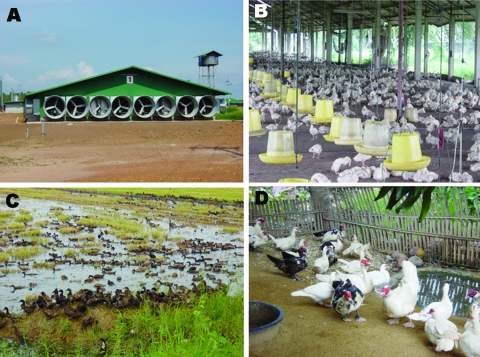
Duck-raising systems in Thailand. A) Closed system with high biosecurity, an evaporative cooling system, and strict entrance control. B) Open system but with netting to prevent entrance of passerine birds. Biosecurity was not strictly enforced. This system is no longer approved for the raising of poultry. C) "Grazing duck raising." Biosecurity is never practiced in this system. D) Backyard Muscovy ducks raised for a family; no biosecurity is practiced in this system.

Four systems were in use during 2004 (Figure 1).

### Closed High-Biosecurity System

Pekin ducks and white Cherry Valley ducks are raised in closed sheds housing 5,000–6,000 birds each. Day-old ducklings are raised for meat in 50 to 55 days by using an "all-in/all-out" system. Before the ducks are sent to slaughter, 60 cloacal samples (≈1%) are collected for virus isolation. In the slaughterhouse, 60 additional samples are collected from the same flock for virologic analysis. At the end of every 50- or 55-day cycle, each poultry house is cleaned and disinfected. After 3 to 4 weeks, the farm is repopulated with day-old ducklings and the cycle is repeated. In 2005, ≈2–3 million ducks were raised in this system.

### Open House System

In the open house system, ducks are raised for meat or as egg layers. The species raised for meat, Pekin and white Cherry Valley ducks, are raised essentially as in the closed-house system with the all-in/all-out strategy. Virologic sampling is conducted as described above. At present ≈1 million to 2 million ducks are being raised in this system. The species raised as egg layers are khaki Campbell, native laying ducks, and a crossbreed of the khaki Campbell and native laying duck. Layer ducks are housed in flocks of 3,000 to 4,000 birds. After they begin laying eggs (at 5 to 6 months of age), these ducks are kept for 12 to 13 months or until they stop laying, at which point they are sent for slaughter. After a short period for cleaning the houses, additional ducks are added as space becomes available. Presently, ≈5 million to 8 million laying ducks are raised in this system in Thailand. Laying ducks are sampled for virologic analysis every 3 months. Influenza-positive flocks are culled.

### Grazing System (Free-range Ducks)

**Figure 2:**
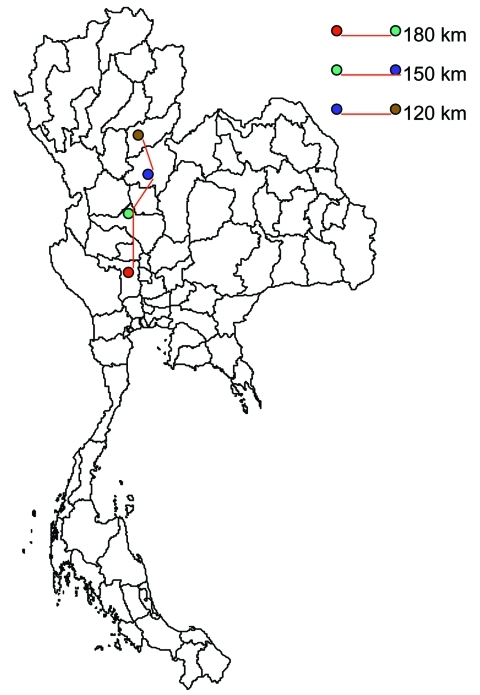
Example of grazing-duck movement. A single flock of ducks was moved 3 times by truck in 1 season in 2004. The size of the flock is 3,000–10,000. The time spent at each site depends on the availability of rice fields at the site: an acre of rice could support 3,000 ducks for 1 to 2 days. The duck owners have agreements with the landowners regarding the time of harvest and the acreage available. One flock could spend as long as 1 month at a single site before being moved to the next.

In 2004, ducks were also raised in the open on rice fields. Most free-range ducks are egg-laying ducks such as khaki Campbell or a crossbreed of khaki Campbell and native laying ducks. However, a small number of "meat" ducks, such as Pekin and white Cherry Valley ducks, are also raised in the open. After hatching and spending 3 weeks in a brooder, young female ducks are moved to rice paddy fields. For the next 5 to 6 months, they grow by eating snails and residual rice after the harvest. When the food supply in 1 field is exhausted, the ducks are moved by truck to another field, often over considerable distances, and even from 1 province to another (Figure 2). When the grazing female ducks are 5–6 months old, they are brought back to the farms, as in the open system described above. However, some flocks of female laying ducks are kept in the rice fields. Male ducks of the species, who are raised with egg-laying hens, and others that are produced for meat are raised in the grazing system for 2 months and are then taken to the slaughterhouses. If they have not reached the optimal weight for slaughter, they are fed supplementary rations for 1 to 2 weeks. During the nationwide surveillance campaign in 2004, 60 cloacal swab samples from each flock were collected for virologic analysis, and the whole flock was culled if a single duck was positive for H5N1 by virus isolation. Flocks that were negative for virus were monitored and put into houses. At the beginning of 2004, ≈10 million to 11 million grazing ducks were being raised in Thailand. Raising free-range ducks is currently illegal in Thailand; all are housed.

### Backyard Ducks

Mixed species of ducks continue to be raised in the backyards of village homes together with other animals, including chickens, geese, and pigs. The duck species raised in backyards include Pekin, white Cherry Valley, Barbary Muscovy, khaki Campbell, native laying ducks, and mule ducks (a sterile crossbreed of Muscovy ducks and native ducks). If a single case of H5N1 infection is detected in a village, all the poultry in the village are culled. Approximately 1.0 million to 1.5 million ducks were raised as backyard ducks at the beginning of the outbreak in 2004; culling reduced that number to <1 million by August 2005.

## National Surveillance Program

In response to the H5N1 influenza outbreaks in 2004, the government of Thailand dispatched teams to villages to identify infected birds and cull flocks in which infection was detected.

### Sample Collection, Histopathology, Virus Isolation, and Serology

During the study period (February to September 2004), our laboratory received 450 sick, moribund, or dead ducks from 25 flocks in the western and central provinces of Thailand. In the detailed studies (Table 1), blood was sampled for serologic analysis by the hemagglutination inhibition (HI) test. All moribund ducks were euthanized, and their internal organs were collected, fixed with 10% buffered formalin, and processed for histopathologic analysis. Additionally, parts of the brain, lung, trachea, intestine, liver, pancreas, kidney, ovary, oviduct, testes, heart, and tight muscle were collected for virus isolation. The tissues were ground and filtered through 0.2–μ filters. The filtrates of each organ were injected into 9- to 11-day-old embryonated chicken eggs and incubated at 37°C for 2 days. The eggs were observed daily to determine whether death occurred. The allantoic fluid was harvested and tested for influenza virus by HI assay. Any positive sample was then subtyped for H5N1. A second egg passage was performed if the embryonated eggs were still alive 72 hours after injection.

**Table 1:** Studies of H5N1 influenza in grazing ducks in Thailand, February to July 2004*

Flock no.	Approximate no. ducks	Age when positive for H5N1 virus (d)	Duration of virus shedding before detection of illness or culling (d)	Highest viral titer (log_10_ EID_50_/mL)	Antibody titers to H5N1 (HI) (log_2_) before culling
1†	4,600	66	8	2.0	<1‡
2§	5,200	78	10	3.1	2
3†	8,000	42	5	2.0	<1
4†	6,800	74	7	2.5	2
5†	4,300	93	5	3.3	2
6¶	7,200	59	5	3.6	2
7¶	10,000	82	7	ND	ND
8†	6,300	60	9	3.8	2
9†	9,800	71	10	ND	ND
10†	5.500	51	6	3.4	ND

*EID_50_, 50% egg infectious dose; HI, hemagglutination inhibition; ND, not done. †Suphanburi Province. ‡Serum samples collected ≈12 d after flock moved to rice field. §Nakornpathom Province. ¶Ayuthdhaya Province.

### H5N1 Subtyping

Avian influenza virus was subtyped by HI assay by using antiserum specific against the H5 hemagglutinin. Reverse-transcription polymerase chain reaction (RT-PCR) analysis was used for H5 and N1 typing (14).

### Immunohistochemical Testing

To evaluate histologic changes, we used immunohistochemical testing by indirect immunoperoxidase staining as described (15). Tissue was fixed in formalin before being embedded in paraffin, then cut in 5–μ-thick sections and mounted onto silanized slides.

### Criteria for Culling Ducks

The criteria for culling duck flocks were based on H5N1 virus isolation and identification by serologic and RT-PCR analysis (12). During the screening of village poultry in 2004, a single positive virus isolation resulted in the culling of all poultry (e.g., chicken, ducks, geese, quail) in the entire village. If serologic evidence of infection was detected, cloacal swabs of 60 ducks in that flock were collected and processed for virus isolation in embryonated chicken eggs.

## Results

### Detection of Influenza Viruses in Different Duck-raising Systems

#### Closed High-Biosecurity System

As mentioned earlier, ≈1% of every duck flock was sampled for H5N1 detection before being sent to slaughter. More than 10,000 ducks were tested during the study period. No virologic or serologic evidence of H5N1 virus infection was detected in the birds raised in this closed system in western Thailand, including Nakornpathom and Kanchanaburi provinces, despite cocirculation of H5N1 influenza viruses in other duck-raising systems in the region.

#### Open House System

Most farms that raised ducks with the open house system are in western Thailand, including the 4 provinces of Nakornpathom, Kanchanaburi, Suphanburi, and Rachaburi. Birds from 17 farms were tested for infection with virus; in birds from 4 (23.5%), infection with the H5N1 virus was detected.

#### Grazing System

In 28 (45.9%) of the 61 free-range duck flocks tested, infection with H5N1 influenza virus was detected. Investigators studied H5N1 infection in 10 flocks of grazing ducks in Ayuthdhaya, Nakornpathom, and Suphanburi provinces between February and July 2004 to determine the biologic and pathologic features of H5N1 infection in the field (Table 1). No virologic or serologic evidence of H5N1 infection was detected in any of the flocks while they were located in the brooding houses. However, after they were moved outdoors to the rice fields, infection with H5N1 influenza was detected in all 10 flocks; the earliest infection was detected 12 days after the ducks left the brooding houses (flock 3, at 42 days of age). The interval between leaving the brooding houses and detection of H5N1 infection was 12–63 days. Of the 10 flocks, 3 (flocks 2, 8, and 9) showed disease signs; only a few birds (<1%) in each flock were clinically affected. However, the interval between initial detection of H5N1 viruses in the flock and culling was 5–10 days, which supports the contention that most ducks in the flocks showed no disease signs.

Serologic evaluation of the flocks showed that low titers of HI antibody were detected before culling, which indicates that an immune response had already begun without disease signs in most birds. Cloacal virus titers in individual ducks showing disease signs before culling were 2.0–3.8 log_10_ 50% egg infectious dose (EID)_50_/mL which shows that virus was being shed in feces (Table 1). Similar virus titers were detected in asymptomatic ducks.

Signs of disease in flocks, 2, 8, and 9 were depression, lethargy, cloudy cornea, and blindness. However, no deaths were observed in the 10 days before culling.

### Backyard Ducks

Of the backyard poultry, chickens were the most frequently infected; 56% of the chicken flocks tested were positive for H5N1 influenza (12). Ducks were the second most frequently infected; 27% of backyard duck flocks were positive for H5N1. During the second wave of H5N1 infection of poultry and humans in Thailand (August–November 2004), 47% of backyard duck flocks were H5N1 positive. During this time, scientists realized that most ducks infected with H5N1 were asymptomatic.

### Pathologic Features

**Figure 3:**
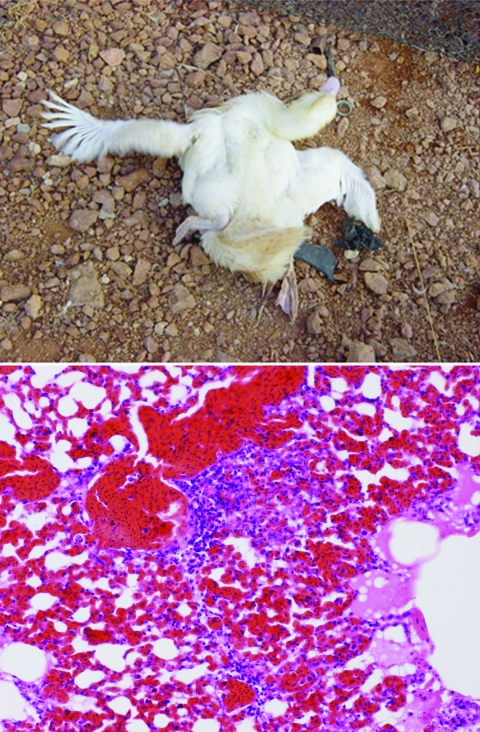
A) A White Cherry Valley duck (*Anas platyrhynchos*), infected with HPAI H5N1 displays nervous signs, convulsions. B) Histopathologic features of the lung of an HPAI H5N1–infected white Cherry Valley duck; infiltration of inflammatory cells in the lung parenchyma (magnification ×100).

As previously mentioned, our laboratory received 450 sick, moribund, or dead ducks, which were studied for pathologic features of H5N1 infection. These birds had been raised in the open house system or were from backyard flocks. They exhibited signs of disease such as high fever, dyspnea, depression, and diarrhea, and nervous signs such as ataxia, incoordination, and convulsions (Figure 3A). Most had ocular and nasal discharge accompanied by conjunctivitis; 20%–100% of the birds in each flock from which these ducks originated were dead. All cloacal and tracheal swabs and tissue samples were positive for H5N1 by HI and RT-PCR (results not shown).

**Figure 4:**
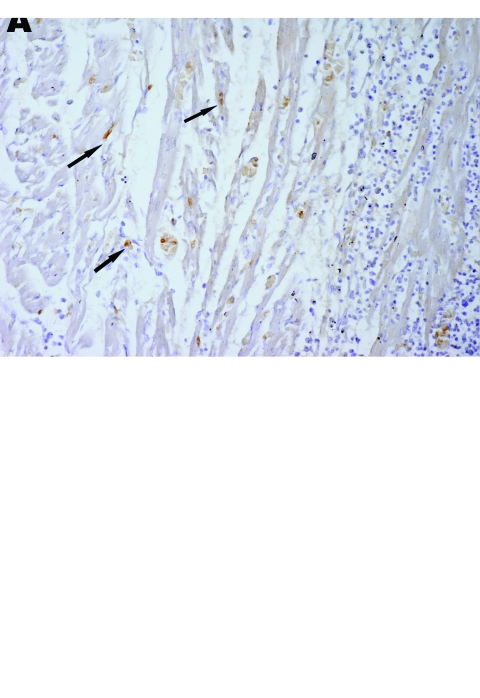
Immunohistochemistry of an HPAI H5N1–infected white Cherry Valley duck (*Anas platyrhynchos*). The viral antigen is detected in myocardial cells and lymphoid cells (arrow) (A) and renal tubular cells (B) (magnification ×100). The primary antibody used for immunohistochemistry in this study was a mouse anti–avian influenza H5 antibody (Magellan Biotechnology, Chunan, Taiwan).

At necropsy, gross lesions were detected, including ecchymotic or petechial hemorrhage of leg and footpad; serous fluid surrounding the heart, pancreas, liver, and abdomen; cyanosis of the oral cavity; and mild pleural effusion. On histopathologic examination, the most striking lesions were found in the lung, with extensive pneumonia and severe pulmonary edema with hyaline material in the alveolar space and slight mononuclear infiltration in the area surrounding congested vessels (Figure 3B). Nonsuppurative encephalitis with perivascular cuffing of mononuclear cells and gliosis were detected in the brains of ducks that displayed nervous signs. Hyaline degeneration and necrosis of myocardium with mononuclear infiltration were detected predominantly in dead ducks from fast-growing breeds such as the Pekin and white Cherry Valley ducks. Necrotizing pancreatis with mononuclear infiltration was detected in all affected ducks. Most affected ducks exhibited focal hepatitis, tubulonephritis, splenic lymphoid depletion or necrosis, and enteritis. Virus antigen was detected by immunohistochemical tests in all organs tested, including trachea, lung, liver, pancreas, rectum, bursa of Fabricius, spleen, brain, heart, and kidney (Figure 4).

### Experimental Infection of Khaki Campbell Ducks

Because culling of all H5N1–positive ducks was mandated in Thailand, we could not determine the natural outcome of infection in birds raised in the open on rice fields. Therefore, khaki Campbell ducks were experimentally infected with 4 representative H5N1 viruses isolated in Thailand in 2004 and 2005. All animal experiments were performed in biosafety level 3+ facilities. All 4 viruses caused the deaths of infected ducks; however, their degree of death varied (Table 2). The most lethal virus tested was A/duck/Thailand/71.1/2004, which caused death in 10/10 of the infected khaki Campbell ducks, a lethality rate comparable to that previously reported for Mallard ducks (16). Also tested was a human virus isolated in 2004, A/Thailand/MK2/2004, which resulted in the death of 2/10 khaki Campbell ducks. Of the two 2005 viruses tested, 1 caused very slight disease and resulted in only 1/10 deaths (A/quail/Thailand/551/2005) whereas the other (A/duck/Thailand/144/2005) resulted in 5/10 deaths. Ducks inoculated with A/Thailand/MK2/04 shed virus for the longest period of time (day 10 postinfection), whereas the 2005 virus isolates were shed only until day 8 postinfection. These results indicate that the H5N1 avian viruses recently isolated in Thailand can cause death in khaki Campbell ducks; however, several infected ducks remained completely healthy with no signs of disease throughout the study.

**Table 2:** Experimental infection of khaki Campbell ducks with viruses isolated in Thailand, 2004–2005

Virus*	Deaths†	Illness†	Day detectable virus was shed‡				
			2	4	6	8	10
A/duck/Thailand/144/05	5/10	3/10	10/10	9/9	1/5	0/5	0/5
A/quail/Thailand/551/05	1/10	2/10	8/10	8/10	1/9	0/9	0/9
A/Thailand/MK2/04	2/10	2/10	9/10	10/10	6/8	2/8	2/8
A/duck/Thailand/71.1/04	10/10	10/10	10/10	1/1	–§	–	–

*Eight 4-week-old khaki Campbell ducks were injected with 10^6^ 50% egg infectious dose of virus by intranasal and intratracheal infection, and 2 contact ducks were introduced 1 day later. †Total number of deaths or birds showing disease/total number of birds. Birds were observed daily for signs of disease or death. ‡Number of ducks shedding by the trachea or cloacae/total number of ducks remaining alive. Ducks were swabbed every other day beginning on day 2 postinfection. §All of the ducks in this group died.

### Current Status of Duck Raising in Thailand

As of October 2005, the government of Thailand forbids the practice of raising ducks in open fields and moving grazing ducks from 1 region to another. Farmers who do so are subject to fines and other punishments. Additionally, they receive no compensation if they raise ducks in the open free-range system, and the ducks become infected with H5N1. Farmers were initially compensated for the culling of their ducks. Duck raising is now confined to the high-biosecurity system.

After a lull of almost 1 year, a case of human H5N1 infection was reported in Thailand in October 2005. The report was preceded by the illegal grazing of 3 flocks of 3,000 to 5,000 free-range ducks in rice fields in the area (Kanchanaburi Province). Although no direct contact between the grazing ducks and backyard chickens was known, within 2 weeks of the arrival of the ducks, chickens in the area began dying, and a person who had direct contact with the diseased chickens died of H5N1 infection. Approximately 500 backyard chickens were culled in the village. Sequence analysis of the human isolate and avian isolates (duck and chicken) from this area would be essential to confirm the epidemiologic link between these cases and, coupled with the chronology of events, to assess whether free-grazing ducks were indeed the source of infection for this outbreak.

## Discussion

The 4 duck-raising systems in wide use at the beginning of the 2004 Thai epidemic differed markedly in cases of influenza detected. No infections with H5N1 influenza virus were detected in ducks raised in the closed system, attesting to the effectiveness of the biosecurity employed. In contrast, H5N1 infection was detected in ducks raised in all 3 open systems. Notably, infection in the hatchery or during the 3 weeks of brooding was detected only after the ducks were released into the rice fields. The source of the H5N1 viruses infecting domestic ducks in the rice fields remains controversial. Because H5N1 viruses were detected in herons, storks, egrets, and other dead waterfowl in Eastern Asia, the initial spread of the highly pathogenic viruses in this region of the world has been attributed to wild migrating birds. What role wild migrating birds had in the spread of H5N1 influenza virus is now a moot question. The widespread outbreaks and massive die-off of bar-headed geese and other species in western China (17,18), and the spread of H5N1 to central Asia (Kazakhstan, southern Russia, and Turkey) and more recently to Romania and Croatia in eastern Europe, are likely caused by wild migratory birds.

Detailed studies of 10 flocks of grazing ducks in Thailand in the present study showed infection with H5N1 influenza virus in all flocks. Although the ducks shed virus for 5 to 10 days, few ducks showed disease signs, and in some flocks, no ducks were symptomatic. Prolonged shedding of H5N1 viruses in experimentally infected ducks has been previously described (16,19), but prolonged shedding in free-range ducks has not. Therefore, free-range (grazing) ducks that are moved long distances by truck and that do not necessarily show disease signs are an optimal vehicle for the spread of H5N1 viruses throughout the country. These findings support the need for regulations that forbid the practice of raising ducks on the free range, a need underscored by the association of the recent human infection with illegal free-range duck grazing.

This study also points out the dangers of raising ducks in the open systems without complete biosecurity. Although stopping the commercial raising of ducks in open system may be impossible, the more problematic issue is that of backyard ducks, which are part of traditional village livestock. Highly pathogenic H5N1 influenza virus is now likely endemic in poultry in Vietnam, Cambodia, China, and Indonesia. The vaccine option should be considered if backyard duck raising is to continue in Southeast Asia.

Although no human cases of H5N1 have been attributed to direct contact with ducks in Thailand, free-grazing ducks have been identified as a risk factor for the occurrence of H5N1 outbreaks among chickens (13). In Vietnam, however, reported human cases of H5N1 influenza have potentially been linked to the consumption of raw duck blood dishes (http://www.who.int/csr/don/2005_01_21/en/index.html). Therefore, H5N1-infected ducks are a risk factor for both commercial and backyard poultry and potentially for humans as well. Since the introduction of the nationwide comprehensive surveillance program ("x-ray surveys") in Thailand (12) and the culling of all infected poultry, human cases of H5N1 infection have been markedly reduced. Traditional methods of duck raising in Thailand and in the rest of Southeast Asia must be modified if we are to control highly pathogenic H5N1 avian influenza.
